# Impact of 4D-Flow CMR Parameters on Functional Evaluation of Fontan Circulation

**DOI:** 10.1007/s00246-024-03446-4

**Published:** 2024-03-22

**Authors:** Lamia Ait Ali, Nicola Martini, Elisa Listo, Elisa Valenti, Julio Sotelo, Stefano Salvadori, Claudio Passino, Angelo Monteleone, Nicola Stagnaro, Gianluca Trocchio, Chiara Marrone, Francesca Raimondi, Giosuè Catapano, Pierluigi Festa

**Affiliations:** 1 Institute of Clinical Physiology, National Research Council, Via Aurelia Sud, 54100 Massa, Pisa, Italy; 2 Gabriele Monasterio Foundation, Pisa, Massa, Italy; 3 Azienda Ospedaliera ASL, 3-Ospedale Villascassi, Genoa, Italy; 4 Departamento de Informática, Universidad Técnica Federico Santa María, Santiago, Chile; 5 IRCCS Istituto Giannina Gaslini, Genoa, Italy; 6 ASST Ospedale Papa Giovanni XXIII, Piazza OMS, 1, 24127 Bergamo BG, Bergamo Italy

**Keywords:** Fontan palliation, Fontan flows, 4D-flow CMR, Cardiac magnetic resonance, Energy loss

## Abstract

We sought to evaluate the potential clinical role of 4D-flow cardiac magnetic resonance (CMR)-derived energetics and flow parameters in a cohort of patients’ post-Fontan palliation. In patients with Fontan circulation who underwent 4D-Flow CMR, streamlines distribution was evaluated, as well a 4D-flow CMR-derived energetics parameters as kinetic energy (KE) and energy loss (EL) normalized by volume. EL/KE index as a marker of flow efficiency was also calculated. Cardiopulmonary exercise test (CPET) was also performed in a subgroup of patients. The population study included 55 patients (mean age 22 ± 11 years). The analysis of the streamlines revealed a preferential distribution of the right superior vena cava flow for the right pulmonary artery (62.5 ± 35.4%) and a mild preferential flow for the left pulmonary artery (52.3 ± 40.6%) of the inferior vena cave-pulmonary arteries (IVC-PA) conduit. Patients with heart failure (HF) presented lower IVC/PA-conduit flow (0.75 ± 0.5 vs 1.3 ± 0.5 l/min/m^2^, *p* = 0.004) and a higher mean flow-jet angle of the IVC-PA conduit (39.2 ± 22.8 vs 15.2 ± 8.9, *p* < 0.001) than the remaining patients. EL/KE index correlates inversely with VO_2_/kg/min: *R*: − 0.45, *p* = 0.01 peak, minute ventilation (VE) *R*: − 0.466, *p* < 0.01, maximal voluntary ventilation: *R*:0.44, *p* = 0.001 and positively with the physiological dead space to the tidal volume ratio (VD/VT) peak: *R*: 0.58, *p* < 0.01. From our data, lower blood flow in IVC/PA conduit and eccentric flow was associated with HF whereas higher EL/KE index was associated with reduced functional capacity and impaired lung function. Larger studies are needed to confirm our results and to further improve the prognostic role of the 4D-Flow CMR in this challenging population.

## Introduction

Fontan is a palliative surgical procedure that allows patients with functionally single ventricle to reach adulthood by directing the systemic venous blood flow into the pulmonary arteries, thus achieving a pulmonary circulation passive filling without the ventricular propulsion [1, 2]. Because of the complexity of the “unnatural” physiology of Fontan intervention, several studies have evaluated the hemodynamics of Fontan by computational fluid dynamics (CFD) [3–5] and in vivo by FOUR-dimensional (4D) flow cardiovascular magnetic resonance imaging (CMR) [6–9].

As a matter of fact, in recent years, the 4D-flow CMR is emerging as an accurate and comprehensive technique for flow evaluation [10, 11]. Moreover, 4D-Flow CMR enables in vivo acquisition of the 3D velocity fields needed to visualize the dynamic 3D blood flow patterns and to quantify novel energetic markers [4]. Recent studies reported a negative correlation between energetic markers and functional capacity in Patients with Fontan circulation [8, 12]. However, there are still few published data on the role of the 4D-Flow CMR in the prognostic stratification of patients with Fontan circulation.

The aim of this study was to evaluate, in patients with Fontan circulation: (1) the flow distribution of the Fontan circuit by the 4D-Flow streamlines, (2) the impact of flow parameters on the adverse outcome with heart failure, (3) the association between the 4D-Flow CMR-derived energetics parameters with lung function and exercise capacity in a subpopulation study.

## Methods

### Population Study

Patients with Fontan palliation who underwent CMR with 4D-Flow acquisition between February 2018 and July 2021 in one tertiary center for clinical indication were included in the study. Exclusion criteria were the presence of atrio-pulmonary Fontan and non-diagnostic 4D-Flow CMR images. The Institutional Review Board of our hospital approved this study, Protocol No. 13756.

Surgical history and clinical data were abstracted from the hospital records: gender, age at CMR, diagnosis, age at Glenn anastomosis if appropriate, age and type of Fontan intervention, and length of follow-up after Fontan palliation. The last echocardiogram report was considered for the evaluation of the degree of atrio-ventricular valve/s regurgitation. Moreover, the following late complications were recorded: cyanosis, arrhythmias, exercise intolerance, protein-losing enteropathy, plastic bronchitis, hepatic, and renal complications.

Fontan patients were considered having overt heart failure if they showed at admission at least one of these manifestations: pleural effusions, ascites, edema, declining albumin, thrombocytopenia, and coagulopathy.

Lung spirometry and Cardiopulmonary test (CPET): CPET was performed on an electrically braked cycle ergometer (Ergostik, Geratherm, Germany) according to ATS/ACCP recommendations [13] in a subpopulation of 34 patients. The protocol included three stages: resting, unloaded pedaling, and exercise and was set to achieve peak exercise in ~ 10 min. The external work rate was continuously incremented using the ramp protocol; CPETs were interrupted when patients reached maximal effort. A CPET with a respiratory exchange ratio (RER) ≥ 1.05 was considered maximal for metabolic stress. A breath-by-breath analysis of expiratory gases and ventilation was performed. Pulse oximetry oxygen saturation was also monitored [14].

We analyzed the following variables: oxygen uptake (VO_2_), and its relationship with heart rate and external work (pulse oxygen or VO_2_/HR and VO_2_/work slope); peak oxygen uptake normalized for the body surface area (VO_2_peak ml/min/kg) CO_2_ production (VCO_2_) and gas ventilatory equivalents (VEVO_2_, VEVCO_2_); the physiological dead space to the tidal volume ratio (Vd/Vt); minute ventilation expressed as the highest value recorded either during exercise or at the first-time recovery phase (VE_peak_); and Maximal Voluntary Ventilation (MVV), Maximal as estimated multiplying FEV1 value by a correction factor of 40 [15].

Baseline Lung function includes the spirometry for static and dynamic lung volume measurements: total lung capacity (TLC); slow vital capacity (SVC); forced vital capacity (FVC); and derived indices as forced expiratory volume in the first second and its percentage of vital capacity (FEV1/VC ratio). Spirometry was performed by experienced technologists. Three spirometric measurements were obtained, and the highest values were chosen in conformity with ATS/ERS standards [14, 16].

### CMR Protocol

A 1.5 Tesla CMR scanner (Signa Artist, GE Healthcare) and 3 Tesla CMR scanner (Ingenia, Philips Healthcare) were used. A comprehensive CMR evaluation was performed following the examination protocol previously published [17].

Briefly, functionally single-ventricle short axis was visualized from the base to the apex, using a cardiac-cine-balanced steady-state free-precession (SSFP) pulse sequence with the following parameters: retrospective ECG gating, field of view 340–360 mm, flip angle 35–50°, TE 1.4–1.9 ms, TR 2.8–3.8 ms, slice thickness 6–8 mm, number of signal averages 1–3, and reconstructed cardiac phases 30. The CMR study was completed using a contrast-enhanced (gadopentetate dimeglumine 0.2–0.4 ml/kg). MR angiographic sequence or a time-resolved angiography for the anatomic evaluation of the Fontan pathway was performed. In patients aged < 8 years or with incapacity to collaborate the CMR exam was performed on deep sedation using titrated propofol.

A 4D-Flow CMR sequence was also prescribed in axial or coronal orientation covering the entire thorax with the following parameters: for both CMR machine: field of view 250–400 mm, TR 3.8–5.3 ms, TE 2.0–3.2 ms, reconstructed cardiac phases 20–32, acquisition time 5–12 min slice thickness 2.2–3.0 mm, VENC according to the velocity in the aorta in the first stage of the study and around 70–100 cm/s subsequently whereas in 1.5 T GE scanner view per segment and flip angle were respectively 2–3, and 13–15 and in 3T Philips scanner turbo field echo (TFE) factor) was 2–3 and flip angle 8–9°.

The SSFP images were evaluated by means of a commercially available software (Mass plus; version 4.0, MR Analytical Software Systems, Leiden, The Netherlands). Ventricular volumes, mass (indexed to body surface area), and the ejection fraction were calculated. 4D-Flow CMR data were processed using Arterys Cardio AI^MR^ (Arterys Inc., San Francisco, CA).

Blood flow quantification was performed by reformatting 4D-Flow CMR data in all Fontan circuit flow; sectional mean wall shear stress, flow eccentricity, and angle jet in the IVC conduit/tunnel-PA conduit were automatically calculated.

Pulmonary arteries and Fontan conduit diameters were measured in axial and latero-lateral planes using multiplanar reformatting of volumetric 3D SSFP in the diastolic phase. The angle between pulmonary arteries and IVC conduit/tunnel-PA conduit was also calculated. Systemic-pulmonary collateral flows (QSPCs) were calculated as: left pulmonary veins flow + right pulmonary veins flow (and) minus right pulmonary artery flow + left pulmonary artery flow [18]; these values were normalized to body surface area. Effective cardiac index (CI) was calculated as (QAo flow − QSPCs)/BSA [19].

Blood flow for pulmonary branches was considered asymmetric if the RPA/LPA flow ratio was > 1.56 (predominant flow for the RPA) or < 0.75 (predominant flow for the RPA) [20].

### Four-Dimensional Flow Magnetic Resonance Imaging Analysis and Blood Flow Energetics

**Fig. 1:**
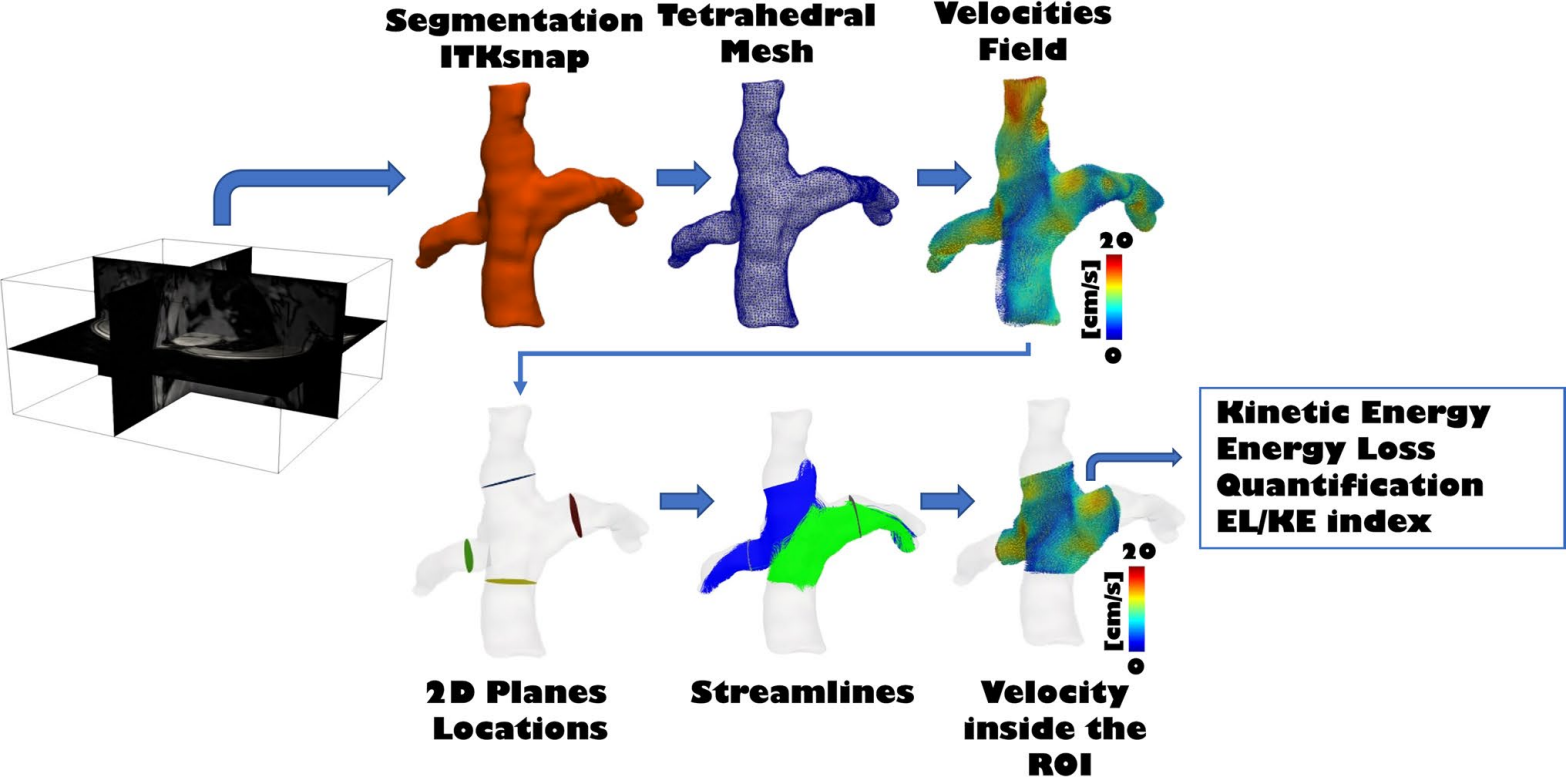
Post-processing 4D-flow MRI data in an extracardiac conduit Fontan patient

The segmentation of the Fontan circuit was manually performed on the phase with the highest contrast on magnitude-weighted velocity images and/or in the phase contrast MR angiography of the 4D-Flow data using open-source TK-SNAP software [21]. Streamlines quantification and visualization were done using ParaView software [22] and were generated using Paraview software, taking as seed points the planes in the SVC and IVC, with a forward integrator type (Runge-Kutta 4-5). 4D-Flow CMR-derived energetics parameters were evaluated in the Fontan confluence including the IVC-Conduit/tunnel-PA, proximal pulmonary branches, and superior vena cava (Fig. 1). The cutting planes were manually placed around 1.5 cm from the bifurcation.

Kinetic energy and energy loss were calculated in a custom-developed software, as previously described [23]. Both parameters were calculated at node level in the 3D domain and then were integrated in a volume of interest, and then normalized by volume (uW/ml) (Fig. 1). KE represents the amount of energy that the blood flow possesses due to its motion; EL represents the amount of KE within the blood flow lost per second due to viscosity-induced frictional forces [3]. In addition, EL index (EL/KE) was calculated as the ratio of normalized EL/normalized KE. EL index (EL/KE) has been described, which can be used as a marker of flow efficiency [7].

### Statistical Analysis

Continuous variables were expressed as mean ± standard deviation (SD) or median (interquartile range IQR: 25th; 75th percentiles) if skewed. Categorical variables were expressed as absolute frequency and percentage.

The correlation between continuous variables was tested with age-adjusted Pearson’s partial correlation coefficient. Leverage points were evaluated in the linear regression analyses: these points were not considered if greater than three times the value of their mean. Age and gender were used as cofactors when appropriate. The effect of gender was evaluated by analyzing the two groups separately. Student’s independent *t*-test was used to compare means between groups of quantitative variables. Statistical analyses performed were considered significant with a *p* value < 0.05. All statistical analyses were performed using SPSS (IBM Corp. Released 2017. IBM SPSS Statistics for Windows, Version 25.0. Armonk, NY: IBM Corp.).

## Results

### Patients Characteristics

From February 2018 to July 2021, 55 consecutive patients [36 males (65%) mean age 22 ± 10 years] were included in the study (40 patients (73%) scanned on 1.5 T GE scanner and 15 patients (27%) scanned on 3T Philips scanner). The most frequent diagnosis in our population was complex atrio-ventricular canal (25%) followed by complex two ventricles (20%), tricuspid atresia and double inlet left ventricle (13%) (Table 1). The single ventricle was morphologically left in almost half of our population (47%). Median age at Fontan procedure was 5.4 (3.8–7) years. Follow-up from Fontan procedure was 14 ± 9 years.

**Table 1:** Population demographic and clinical data

	*n* = 55
Age at the study (years)	21.9 ± 10
Male gender n (%)	36 (65.5%)
Weight (kg) * n* = 55	54.2 ± 18
Height (cm) * n* = 55	159.9 ± 17.9
Body surface area (m^2^)	1.5 ± 0.3
Diagnosis	
Tricuspid atresia	7 (13%)
Double inlet left ventricle	7 (13%)
Complex 2 ventricles	11 (20%)
Hypoplastic left heart syndrome	6 (11%)
Complex Atrio-Ventricular canal	14 (25%)
PAIVS	6 (11%)
Ebstein anomaly	1 (1.8%)
Mitral atresia	3 (5.4%)
Ventricle type	
Two ventricles	8 (14.5%)
Left ventricle	26 (47%)
Right ventricle	20 (36%)
Indetermined ventricle	1 (1.8)
Shunt n (%)	27 (54%)
Glenn n (%)	48 (87.3%)
Age at Fontan (months)	5.4 (3.8, 7.1)
Follow-up from Fontan (years)	14 ± 9
Type Fontan	
Extracardiac conduit	46 (83.7%)
Intracardiac conduit	2 (3.6%)
Lateral tunnel	7 (12.7%)
Fenestrated (%)	17 (31.5%)
Peripheral O_2_ sat (%)	94 (92, 96)
PLE n (%)	4 (7.4%)
Arrhythmia n (%)	9 (17.6%)
Heart failuren (%)	11 (20.4%)
Nt Pro BNP	113 (54, 219)
Significant TR n (%)	22 (44%)
Diuretic therapyn (%)	17 (34%)
Antiarrhythmic therapy n (%)	10 (20.4%)

Continuous variables are expressed as mean ± standard deviation or median and 25°, 75° IQ

*PAVSD* pulmonary atresia and intact ventricular septum; *PLE* protein loose enteropathy

The most common type of Fontan was extracardiac conduit in 84% (*N* = 46); an intracardiac conduit in 2 patients and an intra/extracardiac conduit in one; only 7 patients (13%) had a lateral tunnelt. Clinical and surgical data are summarized in Table 1.

Data from cardiopulmonary test were available in 34 patients (Table 2). CPET was performed in the same day of the CMR study in 21 patients (79.5%), median time between CMR and CPET 0 (IQ 0;0). The population study presents, on the average, a restrictive lung pattern both for volumes and lung diffusion indices (TLC < 80%, DLCO < 75%, table 2) with a degree of muscle deconditioning from reduced transport of oxygen in periphery (Peak VO_2_ml/kg/min = 20 ± 4.9, Oxygen pulse: 9.1 ± 5.6, Table 2). A slight increase in volume/perfusion mismatch was observed in the absence of significant increasing of the physiological dead space ventilation(VE/VCO_2_ slope > 35, peak VD/VT 0.16 ± 0.03, Table 2).

**Table 2:** Lung spirometry and CPET data

Peak VO_2_ (ml/kg/min)	20 ± 4.9
Predicted peak VO_2_ (%)	48.5 ± 10.2
HR reserve (bpm)	58.4 ± 27.3
O_2_ pulse (ml/min)	9.1 ± 5.6
VE/VCO_2_ slope	37.9 ± 8.3
VO_2_ work slope	9.9 ± 1.5
FEV1 (l/min)	3 ± 0.9
FEV 1 (%)	77 ± 19
FEV1/VC (%)	94 ± 11
TL (% of predicted)	79 ± 13.5
Peak VE (l/min)	50 (38.5, 64)
Basal VD/VT	0.22 ± 0.16
Peak VD/VT	0.16 ± 0.03
DLCO sbHB	52.4 ± 13.4
DLCO/VA	71 ± 23
MVV (l/min)	123 ± 28 (117; 42, 64)
BRR (l)	70 ± 28

*BRR* breathing respiratory reserve; *DLCO* the carbon monoxide diffusing capacity; *FEV1* forced expiratory volume in the first second; *MVV* maximal voluntary ventilation; *TLC* total lung capacity; *VC* vital capacity; *VE* ventilation; *DT* dead space; *VA* alveolar volume; *VT* tidal volume

### CMR Data

CMR characteristics of the population study are reported in Table 3. In summary, the median indexed end-diastolic volume was 102 (83–125) ml/m^2^ with a mean ejection fraction (EF) of 53 ± 10.7%. The acquisition time of the 4D-Flow CMR was 7.6 min ± 1.9 min. Flow was slightly higher in RPA than in LPA (respectively 1.2 ± 0.5 l/min/m^2^ vs 1 ± 0.4 l/min/m^2^, *p* = 0.03) with RPA/LPA flow ration of 1.26 (1; 1.6).

**Table 3:** Cardiac magnetic resonance findings

Population study	
CMR	
End diastolic ventricular volume (ml/m^2^)	102 (83, 125)
End systolic ventricular volume (ml/m^2^)	48 (33, 66)
Ventricular ejection fraction (%)	53±10.7
Ventricular mass (g/m^2^)	68.5±19.2
Ventricular mass/volume ratio	0.6 (0.5, 0.7)
SVC flow (l/min/m^2^)	0.9 (0.7, 1.2)
AOPC flow (l/min/m^2^)	0.9 (0.6, 1.5)
Conduit/tunnel IVC-Pas flow (l/min/m^2^)	1.3 ± 0.5
Cardiac index (ll/min/m^2^)	3 ± 0.7
LPA flow (l/min/m^2^)	1 ± 0.4
RPA flow (l/min/m^2^)	1.2 ± 0.5
Left PVs flow (l/min/m^2^)	1.2 (1, 1.6)
Right PVs flow (l/min/m^2^)	1.5 ± 0.4
LPA axial diameter (mm)	13 ± 3.7
LPA latero-lateral diameter (mm)	13.6 ± 3.9
LPA stenosis, *N* (%)	10 (18.2%)
RPA axial diameter (mm)	14.7 ± 4.1
RPA latero-lateral diameter (mm)	15.6 ± 4.4
RPA stenosis, *N* (%)	7 (12.7%)
RSVC_RPA streamlines (%)	62.51 ± 35.37
RSVC_LPA streamlines (%)	38.4 ± 35.31
Conduit/tunnel IVC/Pas_RPA streamlines (%)	47.05 ± 40.50
Conduit/tunnel IVC/Pas_LPA streamlines (%)	52.29 ± 40.65
Mean conduit/tunnel IVC/Pas Vel (cm/s)	31.4 (20.5, 39.7)
Mean conduit/tunnel IVC/Pas Flow jet angle	13.7 (9.9, 23.9)
Mean eccentricity flow conduit/tunnel IVC/Pas	0.05 (0.04, 0.08)

Continuous data are expressed as mean ± SD and or median and IQ.

*CI* cardiac index; *PVs* pulmonary veins; *RPA* right pulmonary artery; *LPA* left pulmonary artery; *SVC* superior vena cava; *IVC* inferior vena cava; *AOPC* aorto-pulmonary collaterals; *RSVC* right superior vena cava

### Caval Flow Distribution

**Fig. 2:**
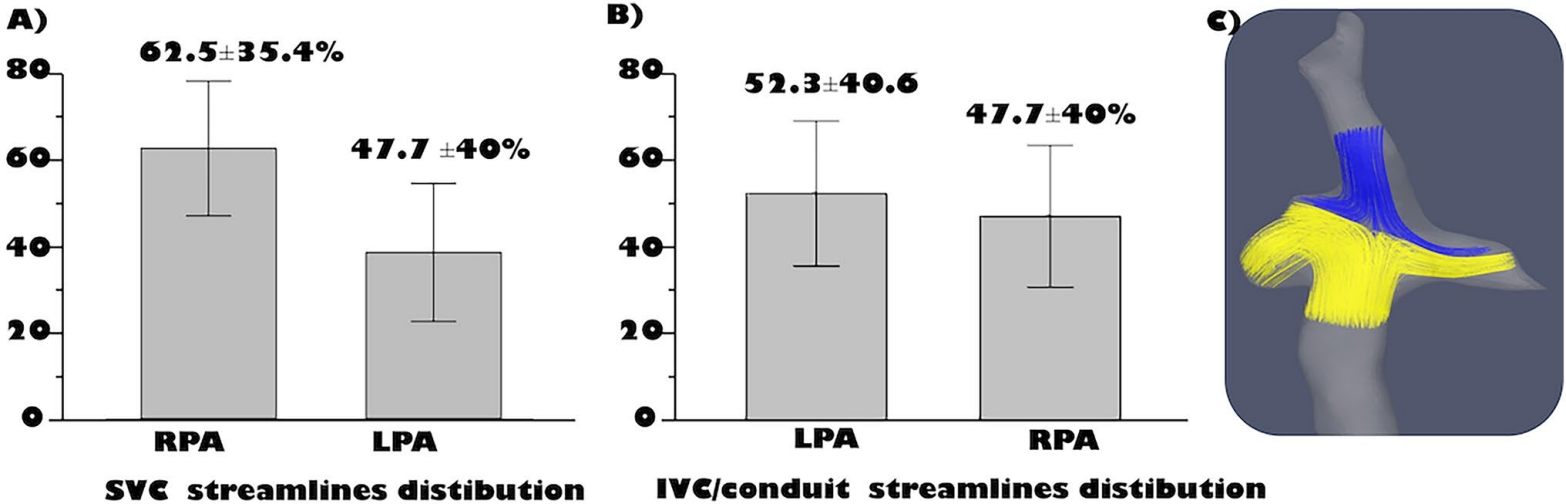
Streamlines flow distribution of the superior vena cava (**A**) and IVC Conduit/tunnel (**B--**) to pulmonary arteries (**B**): **C:** an example of flow distribution in patient with extra-cardiac conduit *LPA* left pulmonary artery; *RPA* right pulmonary artery

The analysis of the streamlines distribution (excluding the 8 patients with persistent left superior vena cava) revealed a preferential distribution of the right SVC flow for the RPA 62.5 ± 35.4% whereas the distribution of IVC-PA conduit flow to the pulmonary branches was quite symmetric with a mild preferential flow for LPA 52.3 ± 40.6% (Fig. 2). However, in 25 patients IVC-PA conduit flow to LPA was more than the 50 %.

### Adverse Cardiac Outcome

Eight patients experienced heart failure (HF) and were admitted at the hospital at the time of CMR study. As expected, in comparison with the remaining population study, patients with HF have a lower peripheral O_2sat_: 85.5 ± 11% vs 94.0 ± 3.1% *p* ≤ 0.001, higher ln-nt proBNP 5.6 ± 0.87 vs 4.48 ± 1.183 *p* = 0.037, and a higher amount Aortopulmonary collaterals flow 0.9 (0.5, 1.3) vs 0.6 (0.3,0.9) *p* = 0.034. Moreover, in HF patients, RPA diameters, indexed IVC/PA conduit/tunnel flow were lower, respectively: 11.3 ± 3.5 vs 15 ± 4 mm, *p* = 0.01, and 0.7 ± 0.5 vs 1.3 ± 0.5 l/min/m^2^, *p* = 0.004, whereas mean sectional jet angle and flow eccentricity were higher, respectively, 39.2 ± 22.8 vs 15.2 ± 8.9 *p* < 0.001 and 0.1 ± 0.04 vs 0.06 ± 0.02, *p* = 0.002 in comparison with the remaining population (Table 4).

**Table 4:** Comparison between HF and non-HF patients

	HF group (*N*: 8)	No HF group (*N*: 47)	*p*
Age at the study (years)	26 ± 18	21 ± 8	0.2
Body surface area (m^2^)	1.52 ± 0.4	1.55 ± 0.332	0.8
Age at Fontan (years)	5.8 (5.3;9.4)	5.3 (3.8;7)	0.543
RPA axial diameter (mm)	11.3 ± 3.5	15.3 ± 4	0.001
RPA latero-lateral (mm)	13.1 ± 4.2	16 ± 4	0.08
RPA stenosis, *N* (%)	3 (37.5)	4 (8.5)	0.02
LPA axial diameter (mm)	13.5 ± 5.9	12.9 ± 3.2	0.706
LPA latero-lateral diameter (mm)	14 ± 5.37	13.54 ± 3.64	0.762
LPA stenosis, *N* (%)	2 (25)	8 (17)	0.6
Conduit/tunnel IVC/Pas flow (l/min/m^2^)	0.75 ± 0.55	1.36 ± 0.55	0.003
SVC flow (l/min/m^2^)	1.27 ± 0.61	0. 99 ± 0.37	0.087
CI 4D (l/min/m^2^)	3.43 ± 0.90	2.97 ± 0.67	0.102
LPA flow (l/min/m^2^)	0.94 ± 0.45	0.98 ± 0.38	0.743
RPA flow (l/min/m^2^)	0.96 ± 0.36	1.25 ± 0.48	0.111
AOPC flow (l/min/m^2^)	1.7 ± 1.47	0.95 ± 0.68	0.030
End diastolic ventricular volume (ml/m^2^)	137 ± 56	105 ± 32	0.026
End systolic ventricular volume (ml/m^2^)	77.5 ± 54.2	51.5 ± 25	0.031
Ventricular ejection fraction (%)	47.6 ± 14.7	54 ± 9.7	0.124
Ventricular mass (g/m^2^)	77.5 ± 28	66 ± 17	0.1
Ventricular mass/volume ratio	0.58 ± 0.12	0.64 ± 0.16	0.3
Peripheral O_2_ sat (%)	85.8 ± 10.4	94 ± 3.1	< 0.001
Ln Nt ProBNP	5.6 ± 0.87	4.48 ± 1.183	0.037
Conduit/tunnel IVC/Pas flow jet Angle	39.2 ± 22.8	15.2 ± 8.9	< 0.001
Conduit/tunnel IVC/Pas Vel (cm/s)	25.4 ± 11	35.2 ± 17.2	0.1
Mean eccentricity IVC/Pas flow	0.1 ± 0.04	0.06 ± 0.02	0.002

The correlation between mean flow jet angle and ln-nt ProBNP values was assessed by using patients' age as the adjustment variable and eliminating leverage points that could incorrectly influence the results. Mean flow jet angle correlates positively with ln-nt Probnp *r* = 0.525, *p* = 0.008.

### Segmental 4D-Flow CMR Energetics Parameters

Norm—Energy Loss, viscous dissipation, and Norm—Kinetic Energy correlate with velocity in the FC respectively (*r*: 0.86 *p* < 0.001, *r*: 0.86 *p* < 0.001 and *r*: 0.98 *p* < 0.001). The effect of patient age was always evaluated in the correlations. The non-significance of the *z*-tests performed to compare the correlation between groups confirmed that gender does not influence the correlations of the variables analyzed.

EL/KE index correlates inversely with BSA *r*: − 0.368, *p* < 0.010, EL/KE index negatively correlated with LPA diameters *r*: − 0.395, *p* = 0.007 and was higher in patients with asymmetric pulmonary blood flow (RPA/LPA flow > 1.56): 0.275 ± 0.07 vs 0.23 ± 0.09 *p* = 0.02.

**Fig. 3:**
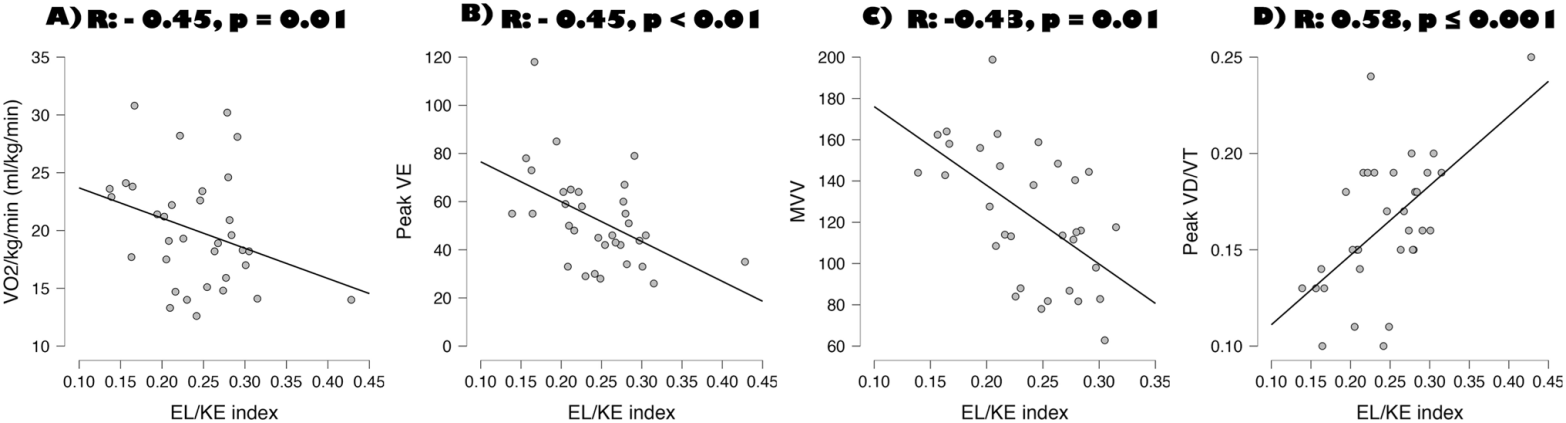
Correlation, corrected by age, of EL/KE index with VO_2_/kg/min (**A**) peak VE (**B**), peak MVV (**C**), (**B**)--- peak and VD/VT (**D**) *KE* kinetic energy; *EL* energy loss; *MVV* maximal voluntary ventilation; *VE* minute ventilation; *VD/VT* physiological dead space to the tidal volume ratio

Moreover, EL/KE index was associated with impaired functional aerobic capacity and reduced ventilation response at physical exercise. As a matter of fact, EL/KE index correlates significantly, inversely with VO_2_/kg/min: *r*: − 0.453 *p* = 0.010, peak VE *r*: − 0.453 *p* < 0.010, MVV − 0.437 *p* = 0.018 and positively with VD/VT peak: *r*: 0.581 *p* < 0.01 (Fig. 3).

## Discussion

Fontan is a surgical palliation in patients with single ventricle physiology by directing the systemic venous return into the pulmonary circulation, achieving passive filling of the pulmonary circulation without ventricular propulsion. Although the long-term survival of patients with Fontan circulation has drastically improved, morbidity is still high [24].

An elevated CVP and reduced cardiac output play an important role in the occurrence of liver fibrosis and decreased exercise capacity [1]. Therefore, surgical construction of an energy-efficient total cavopulmonary connection (TCPC) with low resistance is important to slow down the increase in central venous pressure (CVP) and the decrease of preload towards the systemic ventricle.

CMR is recommended in the follow-up of Fontan patients [24] for the evaluation of ventricular volumes and function, Fontan circulation flows, and their distribution [25] and for quantification of aorto-pulmonary and veno-venous collaterals [19, 26].

Although 2D-flow phase contrast sequence was considered as the gold standard for the flow quantification [25], in recent years, the development of the 4D-Flow volumetric sequence aroused much interest especially for the evaluation of complex congenital heart disease. Moreover, 4D-Flow CMR has recently emerged as a promising non-invasive technique for quantification of in vivo TCPC flow efficiency, through novel parameters able to study the dynamic 3D flow pattern [3].

In this study, we evaluated the clinical impact of flow and energetic parameters-assessed 4D-Flow CMR in patients with Fontan. The main results of our study are

- The flow distribution of the SVC was preferential to the RPA while the flow distribution of IVC Conduit/tunnel-PA was quite symmetric with a slightly higher distribution to the LPA.
- Patients with HF have lower blood flow of IVC Conduit/tunnel-PA and higher mean sectional jet angle and vorticity in comparison with the remaining population.
- Higher EL/KE index was associated with reduced functional aerobic capacity evaluated by peakVO_2_/kg/min and impaired lung function, expressed as reduced ventilatory response (peak VE and MVV) with increased of the dead space ventilation (VD/VT) at physical exercise.

### Distribution of the Fontan Circuit

Considering the quantification of streamlines distribution in the whole population, superior vena cava blood flow was predominantly distributed to the RPA, whereas the distribution of the IVC blood flow was quite symmetrical with a mild preference for the LPA. This is in line with previous publication [9, 27].

#### Hemodynamic Data 4D-Flow CMR and HF

From our data, within the limits of the sample of our population, patients with HF had a reduced flow in the IVC conduit/tunnel-PA and smaller RPA diameters; moreover, we found that a jet angle and eccentricity at the IVC Conduit/tunnel-PA were higher in patients with HF: both parameters could reflect flow eccentricity and vorticity that reduce the flow efficiency [7]. Jet angle and eccentricity, calculated automatically without needing further post processing segmentation, could be interesting parameters but their clinical significance should be confirmed in larger population study.

### Flow Energetics Parameters

Flow energetics of the Fontan circuit were evaluated in the caval veins confluence area, where a higher proportion of KE is dissipated, caused by the confluence of different flows [7]. We focused on EL/KE index that reflects the energy dissipated in comparison with the energy presented due to motion, instead of evaluating EL, which increases with the increase of the blood flow in Fontan circulation [4]. EL/KE index in the Fontan confluence was higher in patients with pulmonary branches stenosis or hypoplastic diameters in line with previous studies that evaluated Fontan hemodynamics by computational fluid dynamics analysis and highlight that undersized pulmonary arteries or Fontan pathway stenosis did have a detrimental effect on power loss [4].

Rijnberg et al. recently found a correlation with both KE and EL in the entire TCP with peak VO_2_ [8]. In our study, we demonstrate that EL/KE correlates with peak VO_2_ and also with respiratory efficiency parameters (MVV; peak VE and peak VD/VT). As a matter of fact, higher EL/KE was associated with lower exercise capacity and impaired lung function with increased lung dead space.

In patients with Fontan circulation, the reduced functional capacity was attributed to lowered ability to increase cardiac output because of a reduction in ventricular preload [1, 28]. However, abnormalities of lung function have been also implicated in the impairment of the exercise tolerance [28, 29] as Fontan hemodynamics is also depending on efficient lung mechanics [29]. As a matter of fact, a non-pulsatile flow could contribute to endothelial changes and dysfunction and an impaired augmentation of venous return may influence the exercise capacity; moreover, exercise in Fontan circulation is not associated with a significant decrease in pulmonary vascular resistance [29, 30] .

The association between the EL/KE index and lung functions parameters raised the question if the impairment of the lung function is a consequence or a cause of the loss of flow efficiency. An increase in wasted ventilation (VD/VT) observed in our study group may be related to a ventilation-perfusion mismatch for high ratio areas from hemodynamic causes (based on pulmonary blood flow efficiency in the Fontan confluence). Furthermore, a restrictive pattern often present in our population (as in previous Fontan cohort), has been attributable to intrinsic lung abnormalities and surgical sequelae [29]. The hypothesis that reduced flow efficiency could have an impact on lung function needs further larger studies.

## Limitations

The study has the limitation of a single-center study with limited sample. The movement of structures along the cardiac phases in the Fontan circulation was not considered in this study due to technical limitations in 4D-flow MRI acquisitions [31]; however, the EL and KE are evaluated inside the lumen and this variable is not evaluated in the arterial wall. Another limitation of the study was the use of data from two different magnetics fields; however, when performing the statistical analysis for 1.5 magnetic field independently, no significant differences were found. Further study with a larger cohort of patients should evaluate the impact of the machine vendor and the magnetic field in the association between 4D-flow advanced parameters and clinical data. Moreover, in the first stage of the study, the velocity encoding was set according to the velocity of aorta. We checked the flow in IVC also calculating the flow in the descending aorta.

## Conclusion

In our cohort of patients with Fontan circulation, lower blood flow in IVC/PA conduit and eccentric flow were associated with HF whereas higher EL/KE index was associated with reduced functional capacity evaluated by CPET, in particular with impaired lung function. Larger studies are needed to confirm our results and further improve the prognostic role of the 4D-Flow CMR in this challenging population.
